# Compliance with the recommended daily intake of at least 400g of fruits and vegetables and its relationship with behavioural change stages in Mexican adults

**DOI:** 10.1017/jns.2025.10058

**Published:** 2025-12-12

**Authors:** Alejandra Jiménez-Aguilar, Rebeca Uribe-Carvajal, Araceli Salazar-Coronel, Cristina Álvarez-Sánchez, Sonia Rodríguez-Ramírez, Ivonne Ramírez-Silva, Teresa Shamah-Levy, Carmen Morales-Ruán

**Affiliations:** 1 Center for Evaluation and Surveys Research, National Institute of Public Health, Cuernavaca, Morelos, Mexico; 2 Nutrition and Early Childhood Development, UNICEF, New York City, USA; 3 Center for Nutrition and Health Research, National Institute of Public Health, Cuernavaca, Morelos, Mexico

**Keywords:** Adults, behavioural stages of change, fruit and vegetable intake, Mexico

## Abstract

Promoting fruit and vegetable (F&V) consumption is a public health priority. This study assessed compliance with the international recommendation of consuming at least 400g of F&V daily among Mexican adults and analysed its association with the five stages of behaviour change from the Transtheoretical Model (Precontemplation, Contemplation, Preparation for action, Action, and Maintenance). Using data from 5203 adults (ages 20–59) in the 2016 National Health and Nutrition Survey, dietary intake was collected via a food frequency questionnaire. Compliance was defined dichotomously (Yes/No), and readiness to change was assessed using a specific survey module. Multiple logistic regression models examined the association between stages of change and compliance, adjusting for demographic, socioeconomic, and health characteristics, as well as perceived barriers and self-efficacy. Nationally, 26.3% of adults met the F&V recommendation. Compliance was significantly higher among individuals in the Preparation for action (OR 3.62, 95% CI: 1.82–7.19), Action (OR 4.50, 95% CI: 1.8–11.25), and Maintenance (OR 9.54, 95% CI: 4.76–19.13) than those in the Precontemplation stage. Higher compliance was also significantly associated with greater self-efficacy (OR 1.86, 95% CI: 1.4–2.47), being in the highest socioeconomic tertile (OR 1.71, 95% CI: 1.25–2.33), and living in the central region (OR 1.70, 95% CI: 1.18–2.45). Conversely, individuals reporting a dislike for vegetables were less likely to meet recommendations (OR 0.67, 95% CI: 0.48–0.94). These findings highlight the value of developing stage-tailored interventions that consider both psychological and structural barriers to improve F&V consumption.

## Introduction

The health and environmental benefits of fruit and vegetable (F&V) consumption are well-documented. A daily intake of at least 400 g of F&V, equivalent to five servings of 80 g each, has been consistently associated with a reduced risk of prevalent non-communicable diseases, such as cardiovascular disease and cancer, as well as a lower risk of micronutrient deficiencies, particularly in vulnerable populations.^(1–3)^

In addition to health benefits, there is an increasing recognition of the role of dietary patterns in environmental sustainability. The EAT-Lancet Commission has called for a global shift toward predominantly plant-based diets to support both human health and environmental goals.^(4)^ In line with this, many national dietary guidelines, including those of Mexico, recommend a diet rich in F&V, with a minimum daily intake of 400 g.^(5)^

Despite these recommendations, global trends show that F&V intake often falls short.^(6,7)^ In Mexico, recent data from the National Health and Nutrition Survey indicate persistently low levels of F&V consumption among adults. Between 2012 and 2022, the average daily intake ranged from 117 to 131 g/day for fruits and 83 to 109 g/day for vegetables. Some populations groups- such as men, younger adults (aged 20–39), individuals living in rural areas or in the northern region of the country, and those with lower socioeconomic status- reported lower median intake levels compared to their counterparts.^(8)^ In parallel, the increasing consumption of ultra-processed foods, accounting for approximately 30% of total energy intake across all groups, may be contributing to the displacement of healthier options like F&V.^(9)^

These trends highlight the urgency of implementing effective strategies to promote F&V consumption and ensure populations meet at least the 400 g a day recommendation.^(1,10)^ Numerous initiatives worldwide have employed behavioural theories to guide such efforts.^(11)^ One of the most widely applied frameworks is the Transtheoretical Model (TTM) developed by Prochaska,^(12)^ which introduces the construct of Behavioural Stages of Change (BSC). This construct posits that individuals exhibit five different levels of motivation, or preparation, for changing their behaviour at different times: (1) Precontemplation, where individuals are not considering change; (2) Contemplation, where they are considering change; (3) Preparation for action, where plans to change are imminent; (4) Action, where recent behaviour changes have been made; and (5) Maintenance, where behaviour change is sustained over time.^(13)^

The BSC construct is central to the TTM and focuses on an individual’s readiness to modify behaviour.^(14)^ These five stages do not necessarily progress in a linear manner, as individuals may move forward or regress depending on factors such as self-efficacy,^(15)^ which is a core concept of the TTM integrated from Bandura’s self-efficacy theory^(16)^ that influences an individual´s ability to progress through the stages of change and maintain behavioural change over time. Its role in increasing confidence, reducing relapse risk, and enhancing motivation makes it a critical factor in interventions on eating behaviour and other health-related changes.^(17,18)^

The temporal dimension of the TTM provides a framework for assessing when and how individuals engage with the change process, making it valuable for designing stage-tailored interventions.^(19,20)^ Evidence suggests that such tailored strategies are more effective in promoting dietary improvements, including increased F&V intake.^(21)^

Given Mexico’s consistently low levels of F&V consumption^(8,22,23)^ and the relevance of behaviour-change frameworks to guide dietary interventions, this study had two objectives: (1) to assess compliance with the international recommendation of consuming at least 400 g of fruit and vegetables per day and (2) to analyse the association between compliance and stages of change as measured by the Fruit and Vegetable Stages of Change module among Mexican adults. The analysis also considered sociodemographic characteristics, psychosocial variables, and health status to identify barriers and facilitators associated with each stage of change, thereby informing strategies to promote healthier eating patterns.

## Materials and methods

### Population and study design

We conducted a cross-sectional observational study using data from the 2016 National Health and Nutrition Survey (2016 ENSANUT). It was carried out from May to October 2016 and is a complex, probabilistic, multi-stage, and multi-topic survey representative at the national, regional, urban, and rural levels.^(24)^ It collects information on the health and nutritional status of the Mexican population across all age groups. This was done through questionnaires, anthropometric and blood pressure measurements, and collection of blood samples from a subsample of the population. The main objective of the 2016 ENSANUT was to quantify the magnitude and distribution of overweight, obesity, and chronic degenerative diseases, and their associated risk factors. The sampling frame of the 2016 ENSANUT in the first stage was a list of basic geostatistical areas (AGEB due to its acronym in Spanish) constructed by the National Institute of Geography and Statistics. AGEBs were stratified according to urbanisation and marginalisation. In the second stage, in the urban AGEBS, six blocks were selected from each AGEB with a probability proportional to their population (registered in the 2010 census). Six dwellings were selected from each block by systematic sampling. In rural AGEBS, three localities were selected with a probability proportional to their population. In each locality, a cluster of 12 dwellings was selected using simple random sampling from a list of clusters constructed by the field team. Once the households were selected, a request was made to speak with the person in charge, and the objective of the 2016 ENSANUT was explained, such as the procedures that would be carried out, and written informed consent was obtained. The 2016 ENSANUT obtained information from 9, 474 households with 29,795 individuals. A questionnaire on the sociodemographic characteristics of family members was administered to each household, and adults (>20 years), adolescents (10–19 years), schoolchildren (5–9 years), and children under 5 years of age were randomly selected. The details of the design and methodology of ENSANUT 2016 have been described elsewhere.^(24)^

This study included men and women aged between 20 and 59 years, with complete information on the variables of interest (*n* = 5419). Because it could have different nutrition as usual, we excluded pregnant or breastfeeding women, given a final subsample of 5,203 Mexican adults (Supplementary Material 1).

### Data collection and variable construction

#### Outcome variable

##### Compliance with the minimum recommended intake of 400g of F&V daily

Dietary data were collected using a previously validated^1^ Semi-Quantitative Food-Frequency Questionnaire (SFFQ) for Adolescents and Adults.^(25)^ The instrument collected data from the last seven days to obtain the current food consumption of the population. It contained 140 food items classified into 14 categories, including a fruit and vegetable group. During the interviews, trained personnel asked respondents to describe their F&V consumption in terms of days of the week, times of the day, portion sizes (based on standardised home measurements), and total number of portions of each food item ingested seven days prior to the interview. To assess F&V consumption, we estimated the net grams consumed per person for each fruit and vegetable by multiplying the number of days by the time of consumption, standardised grammage, number of servings, and edible portions. The methodology for cleaning and processing the SFFQ data has been described elsewhere.^(26)^

We constructed the F&V consumption variable by summing the total grams of F&V consumed per day.^2^ Vegetables used in high-calorie preparations (e.g. pancakes, soups, and creams), candied fruits, syrups, and juices were excluded from the analysis. To assess compliance with the international recommendation of consuming at least 400 g of F&V daily,^(1)^ we categorised individuals into two groups: those who complied (i.e. individuals whose intake was ≥ 400 g/day) and those who did not comply (i.e. intake < 400 g/day).

#### Independent variable

##### Fruit and vegetable stages of change (F&VSC)

Recognising the importance of understanding motivational readiness among the Mexican population, ENSANUT incorporated, for the first time, a Fruit and Vegetable Stages of Change (F&VSC) module into its 2016 national survey. This tool, grounded in the TTM, assesses individuals’ stages of change in relation to meeting the recommended 400 g per day of F&V intake, as well as their perceived self-efficacy.

We created the F&VSC survey module based on the Stages of Change Questionnaire for Exercise (Short Form) designed by Marcus.^(27)^ Perez et al. used this questionnaire to study physical activity in a sample of Mexicans between the ages of 20 and 59 years,^(28)^ similar to our population. Trained personnel administered the five questions included in the F&VSC module. Table 1 shows how we adapted the questions to explore the F&V stage of change in consumption. The response options are listed in the table’s footnote, including the ‘no response’ option, which was recorded by the interviewer when a participant did not choose any of the other options (yes/no/do not know). The Spanish version of the questions is available in Supplementary Material 2. F&VSC was classified into five categories based on participants’ reported behaviours and intentions related to fruit and vegetable consumption as follows: (1) Precontemplation: individuals who reported not consuming F&Vs and expressed no intention to do so; (2) Contemplation: individuals who did not currently consume F&V but intended to begin within the next six months; (3) Preparation for action: individuals who currently consumed F&Vs but not yet the recommended five servings per day; (4) Action: individuals who had recently started consuming at least five servings of F&Vs daily, but for less than six months; and (5) Maintenance: individuals who had been consuming at least five servings of F&Vs daily for six months or more. The operationalisation of each stage is detailed in Table 2, along with the specific questions and response patterns used to assign participants to each category.

**Table 1.** Stages of Change Questionnaire (Short Form) designed by Marcus and adapted to assess F&V stage of change consumption

Original questions^*^	Adapted questions^†^
(1) I currently exercise.	(1) Do you currently eat fruit and vegetables?
(2) I do not currently exercise but intend to do so within the next six months.	(2) If you do not currently eat fruit and vegetables, do you intend to do so within the next six months? ^‡^
(3) I currently exercise regularly.	(3) Do you currently eat at least five fruits and/or vegetables every day?
(4) I have exercised regularly for the past six months.	(4) Have you eaten at least five fruits and/or vegetables daily during the last six months?
(5) In the past, I exercised regularly for at least three consecutive months.	(5) During the past years, have you consumed at least five fruits and/or vegetables daily for at least three consecutive months?

* Answer options for each question: yes/no.

† Answer options for each question: yes/no/do not know/no response. The Spanish version of the questionnaire is provided in Supplementary Material 2.

‡ This question was asked when the answer to Question 1 was ‘No’.

**Table 2.** Algorithm used to classify Mexican adults by F&VSC category

F&VSC description in the context of the study	Question number^†^	Valid Response
1. Precontemplation: Individuals who reported not consuming fruits and vegetables and expressed no intention to do it.	1	No
	2	No
	3	No
	4	No
2. Contemplation: Individuals who did not currently consume F&V but reported intending to do so within the next six months.	1	No
	2	Yes
	3	No
	4	No
3. Preparation for action: Individuals who reported currently consuming F&V, but not yet all five servings.	1	Yes
	3	No
	4	No
4. Action: Individuals who had recently started consuming at least five servings of F&V per day, but for less than six months.	1	Yes
	3	Yes
	4	No
5. Maintenance: Individuals who had been consuming at least five servings of F&V daily for six months or more and reported maintaining this behaviour consistently.	1	Yes
	3	Yes
	4	Yes
	5	Yes

† (1) Do you currently eat fruits and vegetables? (2) If you do not currently eat fruits and vegetables, do you intend to do so within the next six months? (3) Do you currently eat at least five fruits and/or vegetables per day? (4) Have you eaten at least five fruits and/or vegetables per day in the past six months? (5) In the past two years, have you eaten a minimum of five fruits and/or vegetables per day for at least three consecutive months?

#### Covariates

##### Psychosocial variables

In addition to incorporating a F&VSC module, we added psychosocial variables to the survey questionnaire, such as perceived barriers and self-efficacy in F&V consumption (the Spanish version of the questions is attached in Supplementary Material 2). The questions were selected from the literature^(29)^ by a group of health and nutrition experts based on their selection of questions on validated constructs that are widely used in dietary behaviour research.^(30)^ Content validity was ensured by assessing the extent to which each question measured its intended construct. Additionally, clarity and comprehensibility were tested and improved by personnel experienced in conducting surveys. Finally, the reliability of the questionnaire was assessed using the test-retest method and administered on two separate occasions with a 30-day interval between the measurements. Pearson’s correlation analysis yielded a moderate correlation coefficient (r = 0.60), indicating acceptable temporal stability.^(31)^

The construct of ‘perceived barriers’ from the Health Belief Model guided our investigation into the obstacles or challenges that prevent individuals from taking the recommended action.^(32,33)^ We constructed a dichotomous variable (yes or no) for each perceived barrier to adopting a healthy diet, because previous literature has reported that people commonly associate ‘healthy eating’ with F&V consumption,^(34,35)^ therefore, we opted for a broader approach by inquiring about difficulties in following a healthy diet rather than directly asking about obstacles to consuming F&V. The categorisation of barriers was based on a literature review identifying the most common challenges to maintaining a healthy diet all of which were incorporated into the questionnaire.^(36–39)^ The question formulated to explore this point was, ‘Among the following factors, which ones do you think would make it difficult for you to adopt a healthy diet?’ The factors were (a) I dislike the taste of vegetables; (b) I do not have sufficient knowledge to prepare healthy meals; (c) I do not have the support of my family to adopt a healthy diet; (d) I prefer to consume sugary drinks, pastries, sweets, and snacks; (e) I do not have enough time to prepare or consume healthy meals; (f) I do not have enough money to buy fruits and vegetables; and (g) I do not feel motivated to eat healthy food. For self-efficacy, we formulated the following question based on the Health Belief Model^13^ and Social Cognitive Theory.^(40)^ ‘How confident do you feel that you will eat at least five fruits and/or vegetables every day?’ The response options were very confident, confident, somewhat confident, or not confident. The test-retest analysis of the question yielded an acceptable Kappa concordance value of 0.22 (unpublished data). For analytical convenience, we constructed a variable with only two categories: (1) very confident or confident and (2) somewhat confident or not confident.

##### Demographic and socioeconomic variables

A family questionnaire was administered to each household head to explore their demographic traits (sex and age), economic status (household characteristics, property ownership, and household appliances), geographic and social data (area, region of residence, and educational level), and information on morbidity.

We categorised sex as male or female and considered age as a continuous variable and divided it into four categories: (1) 20–29 years; (2) 30–39 years, (3) 40–49 years; and (4) 50–59 years. Educational level was included as a categorical variable and divided into four categories: none (no formal schooling), basic (12 years of schooling, including 3 years of preschool, 6 years of primary, and 3 years of lower secondary), medium (3 years of upper secondary), and professional (≥ 4 years, corresponding to a bachelor’s degree or higher). We considered four geographic regions: North, Central, Mexico City, and South,^3^ and defined urban and rural areas as having populations ≥ 2500 and < 2500, respectively.

To characterise socioeconomic status, we used a previously constructed socioeconomic index based on a principal component analysis of eight variables pertaining to housing and the availability of goods and services (household building materials, number of bedrooms, basic services infrastructure, ownership of a car, television, radio, and refrigerator). We chose as our index the first component, which represented 49.3% of the total variability with a lambda value of 3.95 and broke it into three statistical tertiles (T1, T2, and T3) as cut-off points, where T1 denoted the lowest and T3 the highest socioeconomic level).^(41)^

##### Health status variables

To assess health status, trained personnel measured the height and weight of participants using standardised procedures.^(42,43)^ An electronic scale precise to 100 g (seca) was used to measure weight, whereas height was measured using a stadiometer with an accuracy of 1 mm.^(44)^

We also calculated the body mass index (BMI), defined as the weight in kilograms divided by the square of the height in metres (kg/m^2^),^(45)^ with values between 10 and 58 considered valid data,^(44)^ based on previous literature.^(46)^ Analysis was based on the WHO standards (underweight: <18.5, normal weight: 18.5–24.9, overweight: 25.0–29.9, and obesity: ≥ 30.0).^(45)^ Finally, we analysed morbidity, such as a previous self-reported diagnosis of a non-communicable disease (type-2 diabetes and/or hypertension).

### Ethical aspects

This study was conducted in accordance with the guidelines of the Declaration of Helsinki, and all procedures involving the participants were approved by the Research and Ethics Committee (CI:1401) of the National Institute of Public Health. Written informed consent was obtained from all participants.

### Data analysis

**Figure 1.**
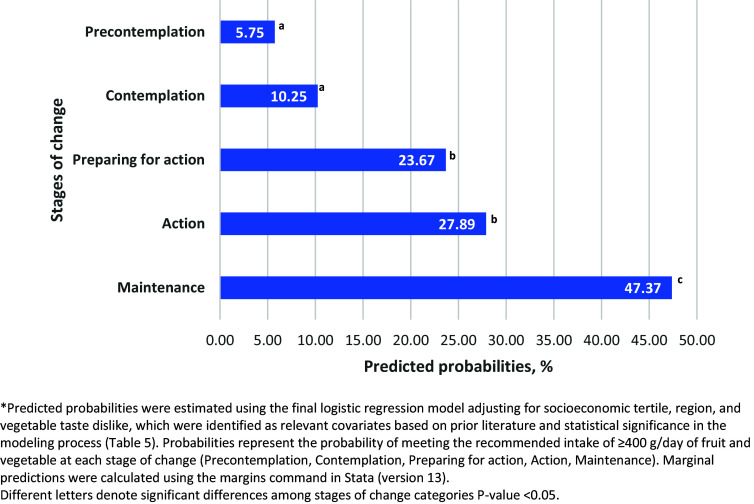
Likelihood* of compliance with the recommended daily intake of F&V (≥400g) according to the stages of change in F&V consumption among Mexican adults.

**Figure 2.**
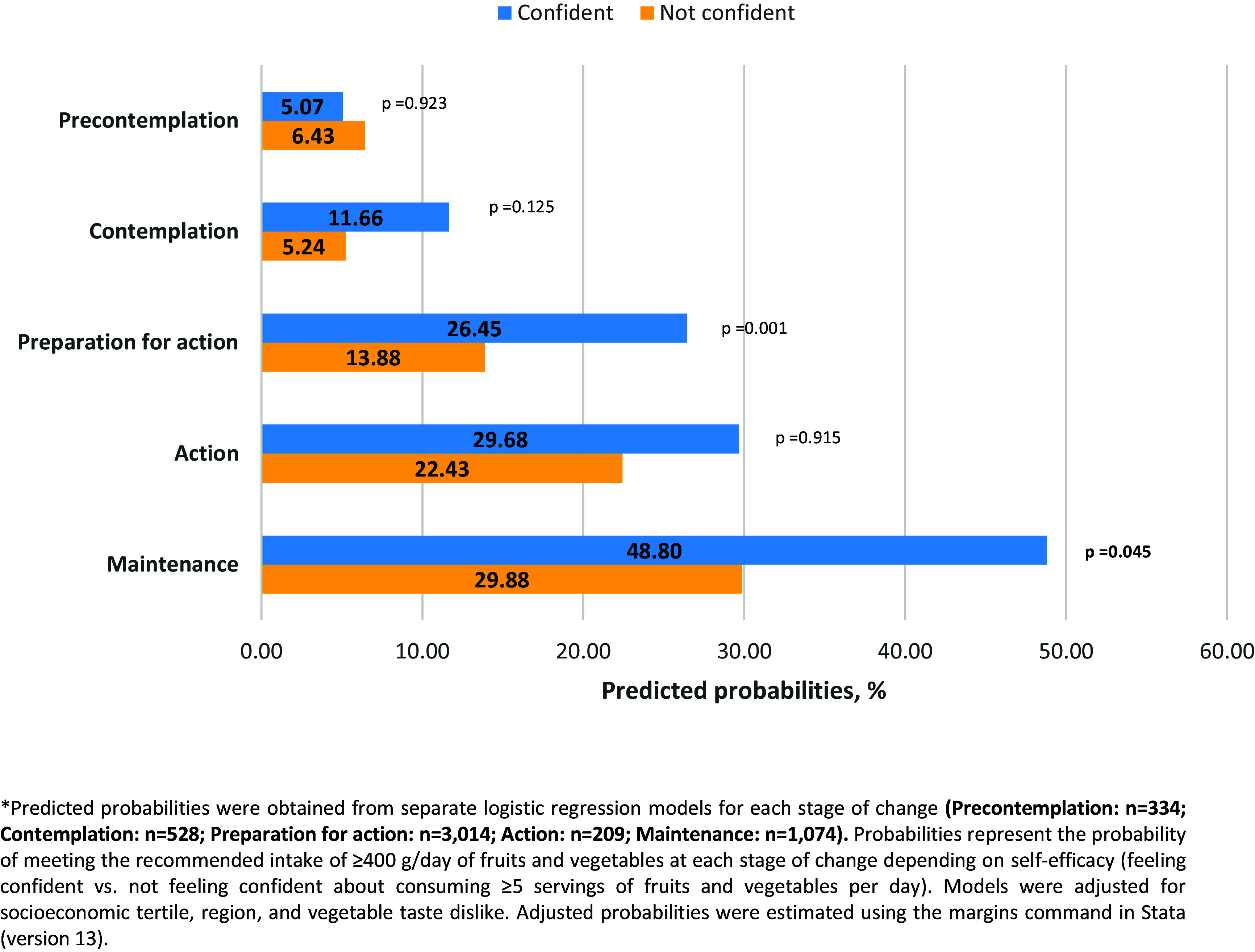
Likelihood* of compliance with the recommended daily intake of F&V (≥400g) according to feeling confident of eating at least five F&V a day and to stage of change in F&V consumption among Mexican adults.

We conducted exploratory analyses of the variables of interest and obtained frequencies and weighted percentages for categorical variables. We calculated the means of the quantitative variables and presented the 95% confidence intervals (CIs) as dispersion measures. To compare variables between men and women, we used the chi-square test for categorical variables and Student’s t-test for continuous variables. Statistical significance was set at *p* < 0.05. We developed multiple logistic models to assess the association between F&VSC (independent variable) and the categories of compliance with the recommended daily consumption of 400 g of F&V (outcome variable). The models were controlled for all variables of interest previously referred to in the literature, such as demographic and socioeconomic characteristics, health status, and morbidity, as well as psychosocial variables, such as perceived barriers and self-efficacy. We tested the interaction terms between the F&VSC and the psychosocial variables. We initially used saturated models (with all variables of interest), and subsequently applied a backward elimination approach to develop a reduced model. This process retained only variables that were statistically significant with a p-value < 0.05.^(47)^ For interaction terms, we used a p-value threshold of ≤ 0.10.^(48)^ The model’s goodness of fit was assessed using the Hosmer-Lemeshow test, and its discriminative ability was evaluated using the area under the ROC curve (AUC). We calculated the adjusted probabilities of compliance with the 400 g daily intake of F&V by F&VSC based on the final logistic regression model. These probabilities were estimated using predictive margins, and are graphically represented in Figure 1. Additionally, to assess the role of self-efficacy in F&V consumption, we conducted five separate logistic regression analyses, one for each stage of change (Precontemplation, Contemplation, Preparation for action, Action, Maintenance). Each model was adjusted for socioeconomic tertiles, regions, and vegetable taste dislike. This analysis evaluated whether feeling confident about consuming at least five F&V per day influenced compliance with the 400 g/day recommendation. The adjusted probabilities for compliance were estimated within each F&VSC and are presented in Figure 2.

All analyses were weighted by expansion factors and adjusted for the survey design using the Stata software (version 13) SVY module for complex surveys (StataCorp, 2013).

## Results

Table 3 presents the characteristics of 5,203 participants, representing more than 42 million Mexican adults aged 20 to 59 years, of whom 52.3% were women. More than half of the survey population was younger than 40, with a mean age of 37.5 years (95% CI: 37.0, 38.1). The respondents resided primarily in the central (33.6%) and southern (29.7%) regions of the country and were predominantly urban (74%). Of the total population, 77% were classified as belonging to socioeconomic tertiles T2 and T3, with 81% having attained a basic or medium educational level.

**Table 3.** Characteristics of participants in the 2016 *ENSANUT*

	National 5,203 (100%)*			Men 1,742 (47.7%)^†^			Women 3,461 (52.3%)^‡^		
Characteristics	*n*	%**	95% CI	*n*	%**	95% CI	*n*	%**	95% CI
**Mean age (years)**	5,203	37.5	(37.0, 38.1)	1742	37.2	(36.3, 38.2)	3461	37.8	(37.2, 38.5)
Age distribution									
20–29	1,137	30.0	(27.6, 32.6)	407	32.8	(28.8, 37.0)	730	27.5	(24.8, 30.5)
30–39	1,500	26.7	(24.4, 29.1)	442	25.5	(22.1, 29.4)	1058	27.7	(25.0, 30.6)
40–49	1,403	24.3	(22.2, 26.5)	461	22.0	(19.3, 25.0)	942	26.3	(23.6, 29.3)
50–59	1,163	19.0	(17.3, 20.9)	432	19.7	(16.9, 22.8)	731	18.4	(16.1, 20.9)
**Mean Body Mass Index (BMI)**	5,203	28.5	(28.2, 28.7)	1742	27.9	(27.5, 28.3)^a^	3461	29.0	(28.7, 29.3)^b^
BMI classification									
Underweight (BMI <18.5 kg/m^2^)	48	1.1	(0.7, 1.7)	15	1.2	(0.6, 2.4)	33	0.9	(0.6, 1.5)
Normal weight (BMI: 18.5–24.9 kg/m²)	1,226	25.5	(23.6, 27.6)	493	27.6	(24.3, 31.2)	733	23.7	(21.4, 26.1)
Overweight (BMI: 25.0-29.9 kg/m²)	1,990	38.9	(36.5, 41.3)	729	42.5	(38.6, 46.4)^a^	1261	35.6	(32.9, 38.5)^b^
Obesity (BMI ≥ 30.0 kg/m²)	1,939	34.5	(32.2, 36.9)	505	28.7	(25.4, 32.4)^a^	1434	39.7	(36.9, 42.6)^b^
**Regions**									
North	1,137	21.0	(18.5,23.8)	379	21.0	(18.2, 24.2)	758	21.0	(17.6, 24.9)
Central	1,735	33.6	(30.8, 36.5)	566	33.8	(30.1, 37.7)	1169	33.3	(30.2, 36.6)
Mexico City and some municipalities in the state of Mexico	561	15.7	(13.4, 18.3)	182	15.9	(13.0, 19.2)	379	15.5	(12.5, 19.2)
South	1,770	29.7	(26.7, 32.9)	615	29.2	(25.9, 32.8)	1155	30.1	(26.6, 34.0)
**Areas**									
Rural	2,698	26.0	(23.4, 28.8)	941	27.4	(24.2, 30.9)	1757	24.8	(22.0, 27.8)
Urban	2,505	74.0	(71.2, 76.6)	801	72.6	(69.1, 75.8)	1704	75.2	(72.2, 78.0)
**Socioeconomic tertiles**									
T1	1,780	23.0	(20.5, 25.8)	640	23.9	(20.4, 27.7)	1140	22.3	(19.6, 25.1)
T2	1,815	30.8	(28.4, 33.2)	592	30.7	(27.3, 34.2)	1223	30.9	(28.1, 33.7)
T3	1,608	46.2	(43.1, 49.4)	510	45.5	(41.0, 50.0)	1098	46.9	(43.1, 50.6)
**Educational level**									
None	313	3.6	(2.9, 4.5)	92	3.2	(2.3, 4.6)	221	4.0	(3.0, 5.2)
Basic	3,567	57.7	(54.8, 60.5)	1169	56.5	(52.3, 60.6)	2398	58.8	(55.1, 62.4)
Medium	908	23.4	(21.3, 25.7)	317	24.5	(21.4, 27.9)	591	22.5	(20.0, 25.2)
Professional	415	15.3	(13.0, 17.9)	164	15.8	(12.8, 19.3)	251	14.8	(11.6, 18.8)
**Previous diagnosis of a non-communicable disease** ^§^									
Type-2 diabetes	417	6.7	(5.7, 7.8)	118	6.1	(4.8, 7.8)	299	7.2	(6, 8.7)
Hypertension	583	10.8	(9.3, 12.4)	147	8.2	(6.3, 10.5)^a^	436	13.1	(11.2, 15.3)^b^
**F&V stages of change**									
-Precontemplation	334	6.5	(5.4, 7.8)	130	7.9	(6.0, 10.3)	204	5.2	(4.1, 6.6)
-Contemplation	528	10.1	(8.7, 11.8)	179	10.1	(8.1, 12.5)	349	10.1	(8.5, 11.9)
-Preparation for action	3,014	56.8	(54.4, 59.2)	989	55.2	(51.4, 58.9)	2025	58.3	(55.4, 61.2)
-Action	209	3.7	(2.9, 4.7)	74	3.6	(2.5, 5.0)	135	3.8	(2.8, 5.1)
-Maintenance	1,074	22.0	(19.9, 24.3)	354	22.0	(18.9, 25.3)	720	22.1	(19.3, 25.2)
-No response	44	0.9	(0.5, 1.4)	16	1.3	(0.7, 2.5)	28	0.5	(0.3, 0.9)

* National population expanded = 42,006,363; ^†^male population expanded=20,035,956; ^‡^female population expanded = 21,970,406

§ *n* = 5,176.

** Percentages are presented except for average age and average BMI, for which their units are specified in parentheses.

Educational level: none (no formal schooling); basic 12 years (3 preschool, 6 primary, and 3 lower secondary); medium 3 years (upper secondary); professional ≥ 4 years (bachelor’s degree or higher).

Different letters denote significant differences by sex according to confidence intervals, and the chi-square test with P-value <0.001.

Women had an average BMI significantly higher (29.0 kg/m^2^; CI95%: 28.7–29.3 kg/m^2^) than men (27.9 kg/m^2^; CI95%: 27.5–28.3 kg/m^2^), and more women were classified as obese (39.7%; CI95%: 36.9–42.6%) than men (28.7%; CI95%: 25.4–32.4%). Likewise, a higher percentage of women reported a hypertension diagnosis (13.1%; CI95%: 11.2–15.3%) compared to men (8.2%; CI95%: 6.3–10.5%). Regarding the distribution of F&V Stages of Change (F&VSC), most respondents (56.8%) were in the Preparation for action stage, followed by Maintenance (22%) and Contemplation (10.1%).

Table 4 presents the distribution of variables related to F&V consumption. Overall, a progressive increase in the mean F&V intake was observed across the stages of change, both at the national level and when stratified by sex, with no notable differences between men and women.

**Table 4.** Compliance and psychosocial variables associated with F&V consumption among participants in the 2016 *ENSANUT*, by sex

Variables	National (5,203)*			Men (1,742)^†^			Women (3,461)^‡^		
F&V stages of change (g/d)	*n*	Mean	95% CI	*n*	Mean	95% CI	*n*	Mean	95% CI
-Precontemplation	334	129.7	(106.2, 153.3)	130	127.6	(92.0, 163.1)	204	132.7	(106.6, 158.8)
-Contemplation	528	182.4	(156.8, 207.9)	179	196.1	(146.3, 246.0)	349	169.7	(150.7, 188.8)
-Preparation for action	3,014	278.5	(263.1, 293.8)	989	281.8	(258.0, 305.6)	2025	275.6	(258.0, 293.2)
-Action	209	295.4	(247.0, 343.7)	74	303.3	(238.8, 367.9)	135	288.6	(224.4, 352.9)
-Maintenance	1,074	443.1	(414.1, 472.1)	354	435.6	(390.2, 481.1)	720	449.8	(411.8, 487.9)
Compliance with 400g daily intake of F&V	*n*	%	95% CI	*n*	%	95% CI	*n*	%	95% CI
Yes	3,951	26.3	(24.2, 28.6)	429	26.4	(23.2, 29.7)	823	26.3	(23.6, 29.1)
No	1,252	73.7	(71.4, 75.8)	1,313	73.6	(70.3, 76.8)	2,638	73.7	(70.9, 76.4)
**Perceived barriers**									
-Dislike for the taste of vegetables	1,132	22.2	(20.3, 24.3)	413	24.3	(20.9, 28.1)	719	20.3	(18.1, 22.6)
-Lack of money to buy vegetables and fruits	3,117	52.4	(49.8, 55.0)	962	48.9	(44.7, 53.1)	2,155	55.6	(52.7, 58.5)
-Lack of knowledge to prepare healthy foods	1,985	38.0	(35.5, 40.6)	670	37.9	(34.1, 41.9)	1,315	38.1	(35.5, 40.8)
-Lack of family support	1,567	32.2	(30.0, 34.4)	503	31.5	(28.3, 35.0)	1,064	32.7	(29.8, 35.7)
-Preference for consuming sugary drinks, pastries, sweets and snacks	1,291	28.7	(26.7, 30.8)	479	30.6	(27.4, 34.0)	812	27.0	(24.5, 29.7)
-Lack of time to prepare or consume healthy foods	1,423	32.7	(30.5, 35.0)	526	35.3	(31.6, 39.1)	897	30.4	(27.8, 33.1)
-Lack of motivation to follow a healthy food	1,367	27.3	(25.1, 29.6)	449	27.3	(23.7, 31.3)	918	27.3	(24.6, 30.0)
-Feeling confident or very confident of eating at least five F&V daily	3,943	78.6	(76.7, 80.5)	1,354	79.4	(76.1, 82.4)	2,589	78.0	(75.7, 80.1)

* National population expanded = 42,006,363; ^†^male population expanded = 20,035,956; ^‡^female population expanded = 21,970,40.

Nationally, 26.3% of the population met the recommended daily intake of 400 g of F&V, indicating that more than seven out of ten adults did not meet the international standard.

In terms of perceived barriers to adopting a healthy diet, 52.4% stated that they lacked sufficient financial resources to buy F&V and 38% reported not knowing how to prepare healthy meals. Approximately 30% of the participants cited barriers such as a lack of time to prepare or consume healthy meals; a lack of family support; a preference for consuming sugary drinks, pastries, sweets, and snacks; and a lack of motivation to eat healthy food, while 22.2% reported disliking the taste of vegetables. In contrast, 78.6% reported feeling confident or very confident that they could eat at least five F&V per day. No significant differences were observed between sexes.

Table 5 presents the results of the final multivariable logistic regression model, adjusted for demographic, socioeconomic, health, and psychosocial characteristics. After the variable selection procedure described in the Methods section, the principal finding was that the odds of compliance with the daily recommended intake of F&V were significantly higher during the Preparation for action, Action and Maintenance stages than during the Precontemplation stage. Likewise, the odds of compliance were significantly higher for individuals in socioeconomic tertile T3 than for those in socioeconomic tertile T1, as well as for people residing in the central region as opposed to the northern region; the odds of compliance were also greater for those feeling confident compared to those who did not feel confident about eating at least five F&V a day. Conversely, disliking the taste of vegetables significantly diminished the odds of compliance. The interaction terms were not statistically significant; therefore, they were excluded from the final model. The model showed a good fit according to the Hosmer-Lemeshow test (χ² = 9.56, *p* = 0.297) and acceptable discriminative ability (AUC = 0.703).

**Table 5.** Relation among compliance with daily recommended intake of F&V (≥400g), stages of change in F&V consumption, socioeconomic tertiles, regions and determinants of F&V consumption*

	OR	95%CI	*P*
Stages of change for F&V consumption			
Precontemplation	1.00		
Contemplation	1.48	(0.61, 3.54)	0.383
Preparation for action	3.62	(1.82, 7.19)	<0.01
Action	4.50	(1.8, 11.25)	<0.01
Maintenance	9.54	(4.76, 19.13)	<0.01
Socioeconomic tertiles			
T1	1.00		
T2	1.10	(0.82, 1.47)	0.542
T3	1.71	(1.25, 2.33)	<0.01
Regions			
North	1.00		
Central	1.70	(1.18, 2.45)	<0.01
Mexico City and some municipalities in the state of Mexico	1.42	(0.95, 2.12)	0.091
South	1.27	(0.89, 1.79)	0.183
No	1.00		
Yes	1.86	(1.4, 2.47)	<0.01
No	1.00		
Yes	0.67	(0.48, 0.94)	0.021

*Adjusted odds ratios (OR), 95% confidence intervals (CI), and *p*-values from the final multivariable logistic regression model, including only statistically significant variables (*p* < 0.05). Analyses were restricted to respondents with complete data for all variables of interest (*n* = 5,112).

Figure 1 shows the adjusted odds of compliance with the daily recommended intake of F&V based on the final logistic model (Table 5). Compliance increased progressively across stages of change, from 5.75% in Precontemplation and 10.25% in Contemplation to 23.67% in Preparation for action and 27.89% in Action, reaching the highest level in Maintenance (47.37%). These results illustrate that individuals in more advanced stages of change were substantially more likely to meet the intake recommendations after adjusting for other variables in the model, such as self-efficacy (feeling confident), dislike of vegetables, socioeconomic tertile, and region.

Figure 2 presents the adjusted probabilities of compliance with the recommended daily intake of F&Vs estimated from separate logistic regression models constructed for each stage of change. The models assessed whether feeling confident about eating at least five servings of F&Vs per day was associated with greater compliance, adjusted for socioeconomic tertile, region, and dislike of the taste of vegetables. The results show that confidence was associated with a significantly higher probability of compliance in the Preparation for action stage (*p* = 0.001) and marginally significant difference in the Maintenance stage (*p* = 0.045).

## Discussion

Our study found that compliance with the international recommendation to consume at least 400 g/day of F&V was significantly associated with individuals’ stages of change. Specifically, individuals in the more advanced stages of change, such as Preparation for action, Action, and Maintenance, had significantly greater odds of meeting the recommendations than those in the Precontemplation stage. Importantly, this analysis assessed whether individuals’ self-reported readiness to change measured through their position within the stages of change translated into actual dietary compliance. The findings showed that individuals closer to or already meeting their F&V intake goals were more likely to comply. This reinforces the theoretical foundation of the TTM, which proposes that behavioural change progresses through successive motivational stages and that individuals with greater readiness are more likely to engage in and sustain healthy behaviours.^(49–51)^ These findings highlight the potential of stage-matched interventions to effectively support F&V consumption by aligning strategies with individuals’ current motivational states.

Similar associations between dietary compliance and stages of change have been documented in previous studies. Van Duyn et al. conducted a study among U.S. adults participating in the 5 A Day for Better Health community studies and found that fruit and vegetable consumption was strongly linked to stage of change, with individuals in the Action and Maintenance stages more likely to meet dietary recommendations.^(52)^ Additionally, Sporny et al. studied a sample of 615 government employees in the northeastern United States and reported a comparable relationship between dietary stage of change and fat consumption, reinforcing the idea that the TTM can be effectively applied to dietary behaviours.^(53)^ In contrast, our study extends this evidence to a nationally representative subsample of Mexican adults aged 20–59 years, thereby addressing the limited generalizability of prior studies restricted to U.S. populations.

Additionally, low compliance with F&V recommendations remains a persistent challenge in Mexico. Using data from the 2006 ENSANUT SFFQ, Ramírez et al. reported a compliance rate of 24.2%, which was comparable to the 26.3% observed in our study.^(23)^ Similarly, Batis et al. analysed 2016 ENSANUT data based on a 24hour recall, found that even among adults who perceived their diet as healthy, only 20% met the 400 g/day recommendation.^(54)^ Furthermore, between 2012 and 2022, fruit and vegetables consumption trends in Mexico have remained consistently below recommended levels.^(8)^

Internationally, the challenge of low F&V consumption extends beyond Mexico. A study by Hall et al. covering 52 low- and middle-income countries found that only 30% of adults consumed sufficient F&V, with compliance rates of 22.4% in men and 21.6% in women. Significant sex differences were observed in only 15 of the countries analysed.^(6)^

Consistent with the findings of other authors,^(6,23)^ we found no significant differences by sex, age, health status, rural-urban area, or educational level in compliance with internationally recommended daily consumption of F&V. However, we found that F&V consumption was related to the socioeconomic level and the region in which the respondents resided. Compliance was positively associated with the highest socioeconomic tertile rather than the lowest and residing in the central region relative to the northern region. This aligns with literature indicating that efforts to promote changes in F&V consumption must consider the social context, finding that even minor improvements in material and social conditions were associated with notable progress in F&V consumption.^(55)^ These factors include enjoying strong social networks, social standards that upheld F&V consumption, access to sufficient food, and less-crowded households.

Additionally, policies such as financial incentives, subsidies for fresh produce, and educational interventions tailored to different stages of behaviour change have been proposed as effective strategies to overcome economic and social barriers to F&V consumption.^(20,56,57)^ Direct economic support such as conditional cash transfer programmes has shown positive effects on dietary behaviour. In Mexico programmes such as Progresa/Oportunidades, which provide financial aid alongside health and nutrition education, have led to improvements in dietary quality.^(58)^ Expanding these programmes to include specific F&V subsidies could further enhance their impact, while tax incentives for healthy food purchases and subsidies for local F&V production may reduce costs and increase affordability.^(59)^

With respect to psychosocial variables, we found two predominant factors associated with dietary behaviour change: self-efficacy boosted compliance with the daily recommended intake of F&V and dislike for the taste of vegetables inhibited F&V consumption. These factors operated independently of the F&VSC categories and sociodemographic characteristics of the population. Similarly, Van Duyn et al., determined that intervention strategies for increasing F&V consumption should focus on the development of individual self-efficacy and regard taste preferences as a key component of change.^(52)^

A previous study documented that self-efficacy could help individuals feel more secure and able to exert control in changing their behaviour.^(60–62)^ Additional studies determined that self-efficacy was a predictor of progression through the stages of change.^(13,28,52,63,64)^ Richert et al., found that individuals with higher self-efficacy are more likely to engage in planning behaviours to increase F&V consumption.^(62)^ Similar associations have been observed among Brazilian adolescents and young adults,^(65)^ reinforcing the idea that confidence in one’s capacity to eat healthily is a relevant factor across different age groups and cultural contexts.

In line with these findings, we analysed the likelihood of compliance by F&VSC category according to the perceived level of self-efficacy on the part of the respondents. Figure 2 illustrates that the adjusted probability of compliance was significantly higher among individuals who felt confident in the Preparation for action stage (*p* = 0.001), and marginally significant in the Maintenances stage (*p* = 0.045), even after adjusting for socioeconomic status, region, and dislike for vegetables. This suggests that at these stages, self-efficacy may serve as a key facilitator of dietary behaviour change, enabling individuals to take action and sustain their behaviour.

In contrast, although positive trends were observed in the Contemplation and Action stages, the differences were not statistically significant. In particular, low compliance among individuals in the Contemplation stage who did not feel confident underscores the need for public health messaging that provides practical, actionable guidance tailored to individuals who are considering but not yet ready to change. These patterns may reflect motivational or cognitive barriers specific to each stage of change, such as lack of knowledge, planning skills, or behavioural intentions, which may interact with self-efficacy. These factors warrant further investigation in future research.

The results observed in our sample could be useful for the planning and evaluation of F&V promotion programmes.^(66)^ A systematic review of TTM effectiveness in multi-behavioural interventions for changing eating habits and levels of physical activity documented that the principal results of the dietary changes were a reduction in fat consumption and an increase in the consumption of F&V.^(67)^ A study of low-income European adults documented a significant increase in F&V consumption by participants in an intervention group that received counselling on dietary behaviour based on the Stages of Change construct (with 4.9 portions consumed). In contrast, the group that received only brief counselling on this topic consumed fewer F&V (0.87 portions), representing a mean difference of 0.62 portions of F&V between groups (CI95%: 0.09-1.13. *p* = 0.021). Similarly, the increase in the number of participants consuming five or more F&V portions per day was greater for those in the dietary behaviour counselling group (42.2%) than for those who received only brief counselling (26.8%), representing a mean difference of 15.4% between the groups (CI95%: 2.52-28.3. *p* = 0.019). The authors concluded that advice on the benefits of eating F&V is more effective when provided in an individualised manner, that is, considering the stages of change of the individuals concerned.^(68)^ Based on these results, public health initiatives could incorporate individualised or small-group counselling sessions that assess an individual’s stage of change and provide tailored stage-specific guidance, particularly addressing motivational and cognitive needs in early stages such as Contemplation. However, future studies are required to evaluate the feasibility, effectiveness, and long-term impact of these strategies.

The strengths and limitations of our study should be considered when interpreting the results. To the best of our knowledge, this is the first study to apply the BSC construct to analyse fruit and vegetable consumption in a nationally representative subsample of Mexican adults aged 20–59 years. However, a significant limitation is the age of the data, which has been almost a decade since they were collected. However, this remains the most recent and nationally representative dataset included in the F&VSC module, so we believe that the findings continue to offer valuable information for understanding behavioural preparedness and informing future public health interventions.

Another limitation is that the F&VSC module combined fruit and vegetable consumption, which prevented us from separately analysing the stages of change for each of these food groups. In addition, apart from the distaste for vegetables, we were unable to identify the determinants for each food group. We jointly investigated the stages of change in F&V consumption to ensure consistency with the international recommendations for five F&V per day. However, according to Glasson et al., health promotion efforts should address the consumption of F&V separately, because knowledge, intake, and accurate perceptions are lower for vegetables than for fruits. Accordingly, they maintained that interventions must place greater emphasis on the consumption of vegetables than fruits.^(69)^ This recommendation was corroborated by our results, which showed that an important barrier to complying with the five-a-day F&V international recommendation was dislike for the taste of vegetables. Therefore, an important improvement in future studies would be to examine the potential differences between barriers to fruit and vegetable consumption separately, allowing for a more nuanced analysis of the specific challenges associated with each food group.

Another limitation is that the survey questionnaire explored perceived barriers previously documented in the literature, leaving open the possibility that other barriers had been omitted. These barriers may be unique to the Mexican population. Therefore, to ensure a more comprehensive understanding of these barriers, we suggest including an open-ended question in future surveys to capture additional factors that have not been previously documented. For instance, financial constraints emerged as a significant barrier in our results, with 52.4% of respondents reporting insufficient resources to purchase fruits and vegetables. Additionally, 38% of the participants indicated a lack of knowledge of how to prepare healthy meals as a key obstacle. Other relevant barriers that could be explored further in the Mexican context include food insecurity^(70)^ and the availability and consumption of ultra-processed foods.^(71)^

Finally, it is important to note that the cross-sectional nature of our survey made it impossible to determine temporality in the relationship between self-efficacy and change from one F&VSC category to another. Nevertheless, as mentioned previously, self-efficacy has been shown to be an accurate predictor of the likelihood of progression from the Action to the Maintenance stage,^(13,28,52,63)^ shedding light on the directionality of the relationship.

Additionally, although both the literature^(52,72,73)^ and our findings support the usefulness of the F&VSC categories for understanding individuals’ readiness to change dietary behaviours, the BSC construct has inherent limitations. A key critique is that categorising individuals into discrete stages may oversimplify the complexity and nonlinear nature of behavioural change. Individuals may move back and forth across stages based on fluctuating motivation, social influences, or structural barriers.^(74)^ Furthermore, the conceptual clarity of the BSC model has been debated, especially concerning the distinctiveness of the stages and the assumption of uniform progression.^(75,76)^ Therefore, we emphasise that the F&VSC module serves as a framework to approximate individuals’ readiness to adopt healthier dietary behaviours rather than as a rigid classification system. To address these limitations, we recommend conducting longitudinal studies in the Mexican population to better understand the temporal dynamics of the transitions between stages. Such studies could provide deeper insights into the factors influencing these transitions and enhance perceptions of self-efficacy^(77)^ ultimately supporting tailored interventions to promote adherence to the five-a-day F&V recommendation, as suggested by Richert et al.

## Conclusions

Our results support the need to implement interventions to improve compliance among Mexican adults with the recommendation to consume five F&V per day. They also support how the Stages of Change construct can be useful in developing more targeted interventions. Similarly, our study shows that interventions will be more likely to be successful if they consider psychological factors, such as self-efficacy and taste preferences, as well as the social context in which the population is concerned. However, future research should prioritise longitudinal approaches to better understand the dynamics of change.

## Supporting information

Jiménez-Aguilar et al. supplementary material 1 Jiménez-Aguilar et al. supplementary material

Jiménez-Aguilar et al. supplementary material 2 Jiménez-Aguilar et al. supplementary material

## Footnotes

1 The FFQ validations was conducted on a random sub-sample of the population selected for the ENSANUT, which had representativeness at the national level and for its four main regions (North, Central and Mexico City, and South) and areas (rural and urban).

2 We included the following F&V in our analysis: avocado, *poblano* chilli pepper, white cabbage, cactus, cucumber, carrot, corn, tomato, chayote, zucchini, broccoli or cauliflower, lettuce, onion, leafy green vegetables, precooked packaged vegetables such as carrot, corn, peas, etc., orange or tangerine, apple or pear, melon or watermelon, peach or apricot, strawberry, guava, jicama, mango, papaya, pineapple, banana, grapefruit and grape.

3 (1) North: Baja California and Baja California Sur, Coahuila, Chihuahua, Durango, Nuevo Leon, Sonora and Tamaulipas; (2) Center: Aguascalientes, Colima, Guanajuato, Jalisco, the State of Mexico, Michoacan, Morelos, Nayarit, Queretaro, San Luis Potosi, Sinaloa and Zacatecas; (3) Mexico City and some municipalities in the state of Mexico, and (4) South: Campeche, Chiapas, Guerrero, Hidalgo, Oaxaca, Puebla, Quintana Roo, Tabasco, Tlaxcala, Veracruz and the Yucatan.

## Supplementary material

The supplementary material for this article can be found at https://doi.org/10.1017/jns.2025.10058
