# Supplementary figures and images for: Compliance with the recommended daily intake of at least 400g of fruits and vegetables and its relationship with behavioural change stages in Mexican adults
Source: J Nutr Sci. 2025 Dec 12;14:e88. doi: 10.1017/jns.2025.10058 (PMC12740495; doi:10.1017/jns.2025.10058)

### Supplementary Material 1. Participant inclusion flowchart

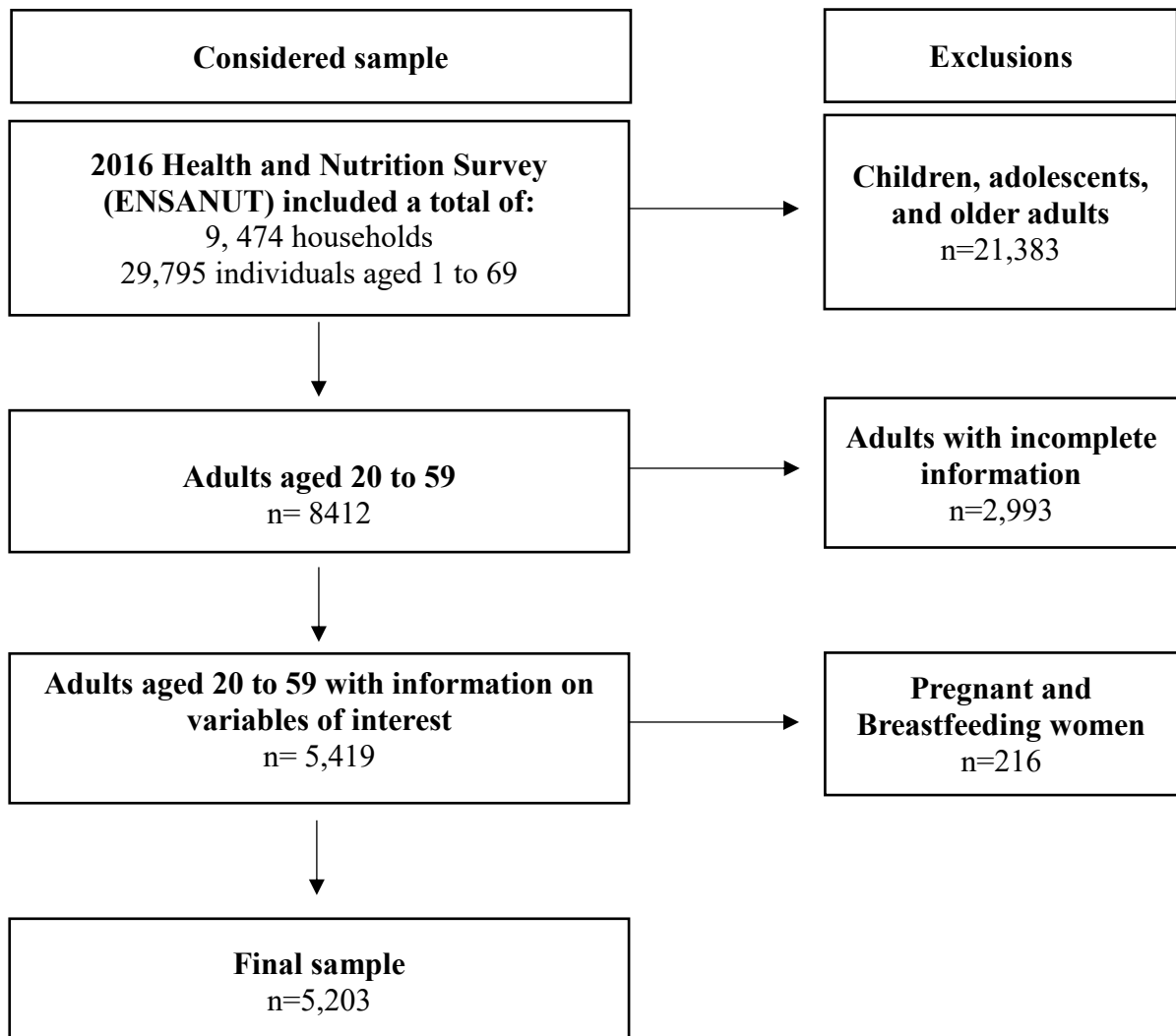

Supplement: Jiménez-Aguilar et al. supplementary material 1 — Jiménez-Aguilar et al. supplementary material [file S204867902510058Xsup001.pdf]
