# Supplementary material for: Compliance with the recommended daily intake of at least 400g of fruits and vegetables and its relationship with behavioural change stages in Mexican adults
Source: J Nutr Sci. 2025 Dec 12;14:e88. doi: 10.1017/jns.2025.10058 (PMC12740495; doi:10.1017/jns.2025.10058)
Supplement: Jiménez-Aguilar et al. supplementary material 2 — Jiménez-Aguilar et al. supplementary material [file S204867902510058Xsup002.pdf]

Instituto Nacional de Salud Pública

ENSANUT Encuesta Nacional de Salud y Nutrición Medio Camino 2016

SALUD SECRETARÍA DE SALUD

Supplementary Material 2. Questionnaire changes in fruit and vegetable consumption in adults in Spanish

<table>
<tr><th>No.</th><th>PREGUNTA</th><th>OPCIONES DE RESPUESTA</th><th></th></tr>
<tr><td>1</td><td>¿Actualmente come frutas y verduras?</td><td>1) Si
2) No
88) No sabe
99) No responde

SÍ= PASE A PREGUNTA 3
NO= PASE A PREGUNTA 2 Y LUEGO A PREGUNTA 5</td><td>|___|</td></tr>
<tr><td>2</td><td>¿Actualmente no consume frutas y verduras, ¿pero tiene la intención de hacerlo dentro de los siguientes seis meses?</td><td>3) Si
4) No
89) No sabe
99) No responde</td><td>|___|</td></tr>
<tr><td>3</td><td>¿Actualmente come por lo menos cinco frutas y verduras al día?</td><td>1) Si
2) No
88) No sabe
99) No responde</td><td>|___|</td></tr>
<tr><td>4</td><td>¿Ha estado comiendo por lo menos cinco frutas y verduras al día en los últimos seis meses?</td><td>1) Si
2) No
88) No sabe
99) No responde</td><td>|___|</td></tr>
<tr><td>5</td><td>En años pasados, ¿ha consumido por lo menos cinco frutas y verduras al día, al menos por tres meses seguidos?</td><td>1) Si
2) No
88) No sabe
99) No responde</td><td>|___|</td></tr>
<tr><td colspan="4">¿Qué tan capaz se siente de…?</td></tr>
<tr><td>6</td><td>…comer por lo menos cinco frutas y verduras al día.</td><td>1) Muy capaz
2) Capaz
3) Poco capaz
4) Nada capaz
88) No sabe
99) No responde</td><td>|___|</td></tr>
</table>

<table>
<tr><th colspan="4">De los siguientes factores dígame cuáles considera que le PUEDEN IMPEDIR llevar una alimentación saludable. Responda “SÍ” o “NO”.</th></tr>
<tr><td>7</td><td>El desagrado por el sabor de las verduras.</td><td>1) Si
2) No
88) No sabe</td><td>|___|</td></tr>
</table>

		99) No responde	
8	La falta de conocimientos para preparar alimentos saludables.	1) Si<br>2) No<br>88) No sabe<br>99) No responde	|___|
9	La falta de apoyo familiar.	1) Si<br>2) No<br>88) No sabe<br>99) No responde	|___|
10	La preferencia por consumir bebidas azucaradas, pastelillos, dulces y botanas.	1) Si<br>2) No<br>89) No sabe<br>99) No responde	|___|
11	La falta de tiempo para preparar o consumir alimentos saludables.	1) Si<br>2) No<br>88) No sabe<br>99) No responde	|___|
12	La falta de dinero para comprar verduras y frutas.	1) Si<br>2) No<br>88) No sabe<br>99) No responde	|___|
13	La falta de motivación.	1) Si<br>2) No<br>88) No sabe<br>99) No responde	|___|
